# Three new species of *Potamothrix* (Oligochaeta, Naididae, Tubificinae) from Fuxian Lake, the deepest lake of Yunnan Province, Southwest China

**DOI:** 10.3897/zookeys.175.2413

**Published:** 2012-03-16

**Authors:** Yongde Cui, Hongzhu Wang

**Affiliations:** 1 State Key Laboratory of Freshwater Ecology and Biotechnology, Institute of Hydrobiology, Chinese Academy of Sciences, Wuhan 430072, China

**Keywords:** *Potamothrix*, Naididae, Tubificinae, taxonomy, new species, Yunnan Province, China

## Abstract

Three new species of *Potamothrix* Vejdovský & Mrázek, 1902 (Oligochaeta: Tubificinae), *Potamothrix praeprostatus* **sp. n.**, *Potamothrix paramoldaviensis* **sp. n.** and *Potamothrix parabedoti* **sp. n.**, are reported from Fuxian Lake of Yunnan Province, Southwest China. *Potamothrix praeprostatusd*iffers from its allies by its prostate glands joining atria in its proximal to middle portion, and spermathecal chaetae. *Potamothrix paramoldaviensis* is distinguishable from its allies by having penial chaeta but no penes, and differs from *Potamothrix moldaviensisb*y its homogenous atrium. *Potamothrix parabedoti* is distinctive in the position of its reproductive organs, and differs from *Potamothrix bedoti* by its homogenous atrium. Hitherto, 34 freshwater oligochaete species have been recorded in Yunnan Province, including nine endemic species from the plateau lakes.

## Introduction

The existence of unique faunae in ancient lakes of Yunnan Province, Southwest China has been recognized in several studies (Yang and Chen 1995; Sket 2000). However, our knowledge of freshwater oligochaetes in these lakes is poor; only an aberrant branchiobdellidan species was reported from Erhai Lake (Liang 1963). During a limnological investigation of lakes in this province in 2002-2003, a number of oligochaete samples were collected. In five previous accounts (Cui and Wang 2005, 2008, 2009, 2012; Cui et al. 2008), 31 species representing 14 genera were reported on the basis of the collected material. As a serial study, this paper gives the description of three new species of *Potamothrix* Vejdovský & Mrázek, 1902 (Oligochaeta: Naididae, Tubificinae) from Fuxian Lake, *Potamothrix praeprostatus* sp. n., *Potamothrix paramoldaviensis* sp. n. and *Potamothrix parabedoti* sp. n.

## Material and methods

Being the deepest lake on the Yunnan-Guizhou Plateau, the Fuxian Lake (24°17'–37'N, 102°49'–57'E) is located in the eastern part of Yunnan Province, and it discharges into the upper reaches of Nanpanjiang River. The lake covers an area of 211 km^2^ at its surface water-level of 1721 m ASL, attaining a maximum depth of 155 m and a shoreline development (D*_L_*) of 1.72. For other characteristics of the lake, the reader may refer to our three previous accounts (Cui et al. 2008; Cui and Wang 2008, 2009).

Lake sediment samples were collected with a weighted Petersen grab (1/16 m^2^) and cleaned with a 250 µm sieve. Large worms were manually sorted in a white porcelain dish and small ones were sorted under a dissecting microscope. Specimens were all preserved in 10% formalin.

Preserved specimens were examined first in temporary glycerine mounts, then stained with borax carmine, dehydrated in an alcohol series, cleared in xylene and mounted in Canada balsam. Measurements of body and chaeta were made from the glycerine mounts. Other observations were made on the permanent mounts. Drawings were made using a camera lucida. Types and other specimens were deposited in Institute of Hydrobiology (IHB), Chinese Academy of Sciences (CAS), Wuhan, China.

### Abbreviation used in the figures

Roman numerals = segment number; at = atrium; mu = muscle; pc = penial chaeta; pe = penis; pr = prostate gland; ps = penial sac; sa = spermathecal ampulla; sc = spermathecal chaeta; scs = spermathecal chaeta sac; sd = spermathecal duct; sf = sperm funnel; sz = spermatozeugmata; vd = vas deferens.

## Taxonomy

### Potamothrix

Vejdovský & Mrázek, 1902

http://species-id.net/wiki/Potamothrix

#### Type species.

*Potamothrix moldaviensis* Vejdovský and Mrázek, 1902

#### Emended diagnosis.

 Hair chaetae present or absent, dorsal chaetae bifid and always pectinated, or only bifids. Ventral chaetae bifids. No coelomocytes. Vas deferens very short, entering atrium apically; atrium tubular, long. Prostate gland small, attached to proximal part of atrium by a short stalk, or no prostate gland. No ejaculatory duct. Penis with or without cuticular sheath. Spermatozeugmata present. Modified spermathecal chaetae present or absent.

#### Remarks.

 The genus *Potamothrix*, established by Vejdovský and Mrázek (1902) for *Potamothrix moldaviensis* Vejdovský & Mrázek, 1902, was revised by Holmquist (1985) and Finogenova and Poddubnaja (1990). Altogether, 20 species were previously known and mainly distributed in the Holarctic region (Table 1) (Brinkhurst and Jamieson 1971; Hrabě 1981; Brinkhurst and Wetzel 1984; Finogenova and Poddubnaja 1990; Šporka 1994; Milbrink 1999; Milbrink and Timm 2001). Through recent investigation of the plateau lakes, three species of *Potamothrix* (Oligochaeta: Tubificinae), *Potamothrix rhytipeniatus* Cui & Wang, 2012, *Potamothrix aductus* Cui & Wang, 2012 and *Potamothrix scleropenis*, have been found in the Fuxian Lake and Xingyun Lake of Yunnan Province, Southwest China (Cui and Wang 2005). They are the lowest-latitude members of the genus hitherto known. Moreover, studies show that *Potamothrix* is unexpectedly species-rich in plateau lakes of Yunnan Province, especially in Fuxian Lake where five species were recorded (Cui 2008; Cui and Wang 2005; Cui et al. 2008). In this paper, we will give the description of three new species, *Potamothrix praeprostatus* sp. n., *Potamothrix paramoldaviensis* sp. n. and *Potamothrix parabedoti* sp. n., from Fuxian Lake.

**Table 1.** Principal distinguishing characteristics of the species of *Potamothrix*

**No**	**Species**	**Chaetae**				**Length ratio of vd/at**	**Prostate gland**	**Atrium**	**Penis**	**Distribution**	**References**
		**Hair**	**Dorsal bifid**	**Spermathecal**	**Penial**						
1	*Potamothrix alatus* Finogenova, 1972	present	pectinated	present	absent or unmodified	1:33–35	present	tripartite	present	Russia	Finogenova and Poddubnaja 1990
2	*Potamothrix bavaricus* (Oschmann, 1913)	present	pectinated	present	unmodified	1:8–9	absent	tripartite	present	Holarctic, Australia, New Zealand	Timm 1970; Brinkhurst and Jamieson 1971; Finogenova and Poddubnaja 1990
3	*Potamothrix bedoti* (Piguet, 1931)	present	pectinated	present	unmodified	1:25–30	absent	tripartite	present	Europe, North America, China	Timm 1970; Timm 1999; Wang and Liang 2001
4	*Potamothrix caspicus* (Lastočkin, 1937)	absent	bifid	absent, or 2–3 bifids	absent or unmodified	1:22–26	present	bipartite	present	Russia	Finogenova and Poddubnaja 1990
5	*Potamothrix cekanovskajae* Finogenova, 1972	absent	bifid	absent, or 4–5 bifids	unmodified	1:28–31	absent	bipartite	present	Caspian Sea	Finogenova and Poddubnaja 1990
6	*Potamothrix danubialis* (Hrabĕ, 1941)	absent	bifid	present	absent or unmodified	1:15–17	present	bipartite	present	Russia	Finogenova and Poddubnaja 1990
7	*Potamothrix hammoniensis* (Michaelsen, 1901)	present	pectinated	present	absent or unmodified	1:40–45	present	bipartite	present	Holarctic	Finogenova and Poddubnaja 1990
8	*Potamothrix heuscheri* (Bretscher, 1900)	present	pectinated	present	unmodified	1:20	absent	tripartite	present	Europe, Israel	Brinkhurst and Jamieson 1971; Finogenova and Poddubnaja 1990; Milbrink 1999
9	*Potamothrix isochaetus* (Hrabĕ, 1931)	absent	bifid	present	-	-	present	-	present	Europe	Brinkhurst and Jamieson 1971
10	*Potamothrix manus* Finogenova, 1972	absent	bifid	present	unmodified	1:14–17	absent	bipartite	present	Caspian Sea	Finogenova and Poddubnaja 1990
11	*Potamothrix marzeki* (Hrabĕ, 1941)	absent	bifid	absent, or 1–2 bifids	absent or unmodified	1:22–24	present	bipartite	present	Russia, Czech	Hrabě 1981; Finogenova and Poddubnaja 1990
12	*Potamothrix moldaviensis* Vejdovský & Mrázek, 1902	absent	bifid	present	unmodified	1:20–32	absent	tripartite	present	Holarctic	Brinkhurst and Jamieson 1971; Finogenova and Poddubnaja 1990; Milbrink and Timm 2001
13	*Potamothrix ochridanus* (Hrabĕ, 1931)	present	bifid	absent	unmodified	-	present	-	present	North America, Serbia	Brinkhurst and Jamieson 1971
14	*Potamothrix orientalis* (Černosvitov, 1938)	present	bifid	present	-	-	absent	-	-	North America	Brinkhurst and Jamieson 1971
15	*Potamothrix postojnae* Karaman, 1974	present	pectinated	absent	-	-	present	homo-geneous	-	Slovenia	Brinkhurst and Wetzel 1984
16	*Potamothrix prespaensis* (Hrabĕ, 1931)	present	bifid	present	-	-	absent	-	present	Serbia	Brinkhurst and Jamieson 1971
17	*Potamothrix svirenkoi *Lastočkin, 1937	present	bifid	present	-	-	present	-	-	Europe, Russia	Brinkhurst and Jamieson 1971
18	*Potamothrix thermalis* (Pop, 1968)	present	pectinated	present	unmodified	1:20	present	tripartite	present	Romania	Pop 1976
19	*Potamothrix tudoranceai* Šporka, 1994	present	pectinated	present	-	1:34	absent	homo-geneous	present	Africa	Šporka 1994
20	*Potamothrix vejdovsky* (Hrabĕ, 1941)	present	bifid	present	absent or unmodified	1:30–33	present	bipartite	present	Europe, North America	Finogenova and Poddubnaja 1990

“-”Unmentioned in the original descriptions

### Potamothrix praeprostatus sp. n.

urn:lsid:zoobank.org:act:A45887B2-F06C-4F6C-B66E-A6F65DACD01B

http://species-id.net/wiki/Potamothrix_praeprostatus

#### Holotype.

IHB YAN 20021205b, mature specimen mounted in Canada balsam, and stained with borax carmine.

#### Type locality.

 East of Lichang (24°32'04"N, 102°51'43"E) in Fuxian Lake, eastern Yunnan, China; depth 113 m, bottom temperature 13.5°C, dissolved oxygen at bottom 5.2 mg/L, total nitrogen in water 0.164 mg/L, total phosphorus in water 0.037 mg/L, fine clay; Dec 11, 2002, coll. Y. Cui and X. Liu.

#### Etymology.

“*prae*” and “*prostatus*” are Latin for “proximal” and “prostate”, respectively. The specific name refers to the prostate glands proximally attached to atria.

#### Description.

 One complete specimen 7.6 mm long, diameter at XI about 0.8 mm, 27 segments. Prostomium conical. Clitellum inconspicuous.

**Figure 1.**
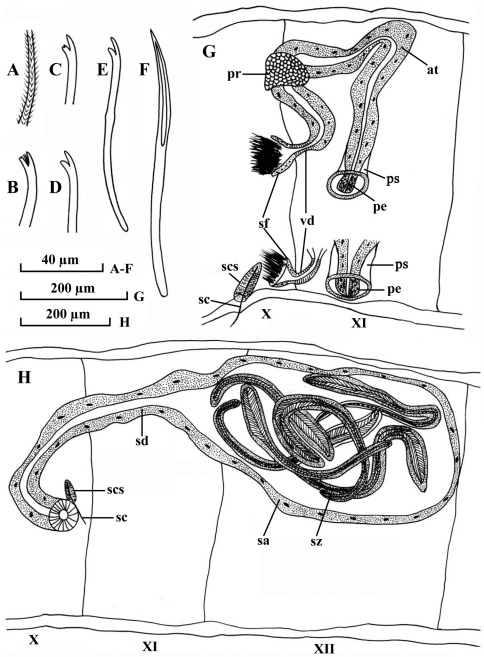
*Potamothrix praeprostatus* sp. n., **A** hair **B** distal end of dorsal bifid from V **C** distal end of dorsal bifid from III **D** distal end of ventral chaeta from V **E** ventral chaeta from III **F** spermathecal chaeta **G** lateral view of male ducts in segments X–XI **H** lateral view of spermatheca in segments X–XII. Scale bars: **A–F** 40 µm; **G–H** 200 µm.

Dorsal chaetae (Fig. 1C–D) of II–IV bifid only, 7–10 per bundle, 135–148 µm long, 3.0–3.5 µm thick, upper tooth longer and thinner than lower, lower tooth occasionally bifurcated. Dorsal bundles of V–X with 5–8 hair chaetae and 5–7 bifid chaetae; plumose hair chaetae (Fig. 1A), 240–420 µm long, 2.6–3.2 µm thick basally; pectinate bifid chaetae (Fig. 1B), 120–140 µm long, 2.8–3.2 µm thick, with 1–2 intermediate teeth, upper tooth slightly longer and thinner than lower tooth (usually bifurcated), or equally long. Dorsal bundles in posterior segments with 1–4 hair chaetae and 2–6 bifid chaetae, shorter and thinner than those of anterior segments, hair chaetae 280–320 µm long, bifid chaetae 90–110 µm long, 2.6–2.8 µm thick. Ventral chaetae (Fig. 1D–E) bifid, 6–8 per bundle anteriorly, 140–150 µm long, 3.0–3.5 µm thick; 2–4 (5) per bundle in postclitellar segments, 80–110 µm long, 2.4–3.2 µm thick, all with teeth similar to the ones in dorsal chaetae in II-IV. Spermathecal chaetae (Fig. 1F, H, sc) one per bundle in middle to posterior of X, entally embedded in glandular sacs, about 145–160 µm long, 4.0 µm thick, with ectal part grooved. Penial chaetae absent. Male pores paired in line with ventral chaetae, anterior to middle of XI. Spermathecal pores paired in line with ventral chaetae, posterior to middle of X, immediately anterior to spermathecal chaetae.

Pharyngeal glands in II–III. Chloragogen cells from VI onwards. No coelomocytes. Male genitalia (Fig. 1G) paired. Vasa deferentia (Fig. 1G, vd) 38–65 µm long, 16–22 µm wide, entering atria apically. Atria (Fig. 1G, at) 690 µm long, 28–80 µm wide, tubular and rather homogenous throughout, with thin outer muscular layer and thick inner epithelium. Prostate glands (Fig. 1G, pr) small, proximally attached to atria, and far from vasa deferentia. Soft part of penis (Fig. 1G, pe) small, 38–54 µm long, 22–44 µm wide, cylindrical, enclosed in penial sacs. Penial sacs (Fig. 1G, ps) 65–80 µm long, 54–80 µm wide, with muscular layer 3–4 µm thick.

Spermathecae (Fig. 1H) in X–XII, ducts (Fig. 1H, sd) 470–490 µm long, 38–65 µm wide, ampullae (Fig. 1H, sa) elongated, 520–540 µm long, maximally 300–315 µm wide. Spermatozeugmata (Fig. 1H, sz) 5–8 in each ampulla, about 300–460 µm long.

#### Distribution and habitat.

Known only from its type locality, Yunnan Province, China; freshwater lake, 113 m depth, water temperature less than 14 °C, fine clay.

#### Remarks.

 According to short vasa deferentia, long tubular atria, each with a small prostate gland, and lack of ejaculatory ducts, the new species fits more closely the definition of *Potamothrix* Vejdovský & Mrázek, 1902 than that of any other described tubificine genus (Brinkhurst and Jamieson 1971; Finogenova and Poddubnaja 1990).

**Figure 2.**
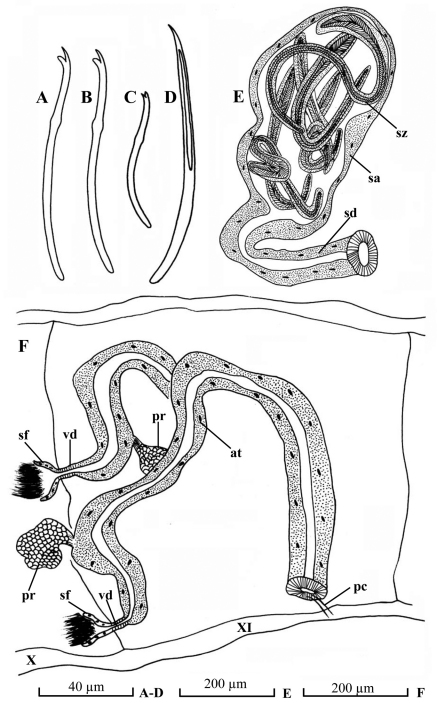
*Potamothrix paramoldaviensis* sp. n., **A** dorsal chaeta from III **B** ventral chaeta from VII **C** penial chaeta **D** spermathecal chaeta **E** spermatheca **F** lateral view of male ducts in segments X–XI. Scale bars: **A–D** 40 µm; **E–F** 200 µm

*Potamothrix praeprostatus* sp. n. differs from its allies by its prostate glands joining atria in their proximal to middle portion. With regard homogenous atria with prostate glands, the new species is similar to *Potamothrix postojnae* Karaman, 1974, *Potamothrix scleropenis* Cui & Wang, 2005, *Potamothrix aductus* Cui & Wang, 2012, and *Potamothrix paramoldaviensis* sp. n. However, these species differ from *Potamothrix praeprostatus* sp. n. in that*Potamothrix postojnae* has no spermathecal chaeta (Brinkhurst and Wetzel 1984); *Potamothrix scleropenis* has penial sheath (Cui and Wang 2005); *Potamothrix paramoldaviensis* sp. n. has no hairs and no penis (Fig. 2); *Potamothrix aductus* Cui & Wang, 2012 has no spermathecal duct and its spermathecal chaeta has contorted ectal part.

### Potamothrix paramoldaviensis sp. n.

urn:lsid:zoobank.org:act:9FE88E3F-B244-443B-9E5D-0DB70AB4559B

http://species-id.net/wiki/Potamothrix_paramoldaviensis

#### Holotype.

IHB YAN 20020812i, mature specimen mounted in Canada balsam, and stained with borax carmine.

#### Type locality.

 East of Gushan Island (24°24'05"N, 102°52'45"E) in Fuxian Lake, eastern Yunnan, China; depth 78 m, bottom temperature 15.9 °C, dissolved oxygen at bottom 9.6 mg/L, total nitrogen in water 0.155 mg/L, total phosphorus in water 0.0234 mg/L, fine clay; Aug 8, 2002, coll. Y. Cui and X. Liu.

#### Etymology.

Named “*paramoldaviensis*” for its resemblance with *Potamothrix moldaviensis* Vejdovský & Mrázek, 1902 in terms of its male genitalia.

#### Description.

 Specimen incomplete, length > 4.4 mm, diameter at XI about 0.7 mm, segments > 13. Clitellum inconspicuous.

Chaetae (Fig. 2A–B) all bifid, 4–6 per bundle dorsally, 3–6 per bundle ventrally, 80–120 µm long, 2.0–2.6 µm thick, upper tooth longer and thinner than lower. Spermathecal chaetae (Fig. 2D) one per bundle in posterior to middle of X, entally embedded in glandular sacs, 145–160 µm long, 4.0–4.5 µm thick, with curved ental part, and grooved ectal part. Penial chaetae (Fig. 2C, F, pc) slightly different to other ventral chaetae, 1–2 per bundle in postero-XI, 70–74 µm long, 2.0–2.4 µm thick, upper tooth as long as, but thicker than lower tooth. Male pores paired in line with ventral chaetae in postero-XI, immediately anterior to penial chaetae. Spermathecal pores paired in line with ventral chaetae in posterior to middle of X, immediately anterior to spermathecal chaetae.

Pharyngeal glands in II–III. Chloragogen cells from VI onwards. No coelomocytes. Male genitalia (Fig. 2F) paired. Vasa deferentia (Fig. 2F, vd) very short, 27–38 µm long, 16–20 µm wide, entering atria apically. Atria (Fig. 2F, at) 1050–1130 µm long, 38–90 µm wide, tubular and rather homogenous throughout, with thin outer muscular layer and thick inner epithelium. Prostate gland small, attached proximally to atrium. Penis absent.

Spermathecae ducts (Fig. 2E, sd) 345–360 µm long, 38–70 µm wide, ampullae (Fig. 2H, sa) pear-shaped, 420–430 µm long, maximally 230–250 µm wide. Spermatozeugmata (Fig. 2H, sz) 6–9 in each ampulla, about 300–640 µm long.

#### Distribution and habitat.

Known only from its type locality, Yunnan Province, China; freshwater lake, 78 m depth, water temperature less than 16 °C, fine clay.

#### Remarks.

According to very short vasa deferentia, long tubular atria each with a small prostate gland, and lack of ejaculatory ducts, the new species fits more closely the definition of *Potamothrix* Vejdovský & Mrázek, 1902 than that of any other described tubificine genus (Brinkhurst and Jamieson 1971; Finogenova and Poddubnaja 1990).

This new speciesresembles *Potamothrix moldaviensisin* some aspects of the male organs (Vejdovský and Mrázek 1902), e.g. the very short vasa deferentia, tubular atria, and the length ration of the vasa diferentia to the atria, and their differences are obvious. *Potamothrix paramoldaviensis* sp. n. has homogenous atria with prostate glands and no penes, while *Potamothrix moldaviensis* has tripartite atria without prostate glands, with short penes.

The new species is distinguishable from other species from the Yunnan lakes in the characteristics of some somatic chaetae. For instance, hair chaetae and pectinate bifid chaetae are present in *Potamothrix scleropenis* Cui & Wang, 2005, *Potamothrix rhytipeniatus* Cui & Wang, 2012, *Potamothrix aductus* Cui & Wang, 2012, *Potamothrix praeprostatus* sp. n. and *Potamothrix parabedoti* sp. n., but hair chaetae are absent in *Potamothrix paramoldaviensis*; the spermathecal chaetae of these six species are dissimilar; slightly modified penial chaetae are present in *Potamothrix scleropenis* and *Potamothrix paramoldaviensis*, but are absent in the other three species (Table 2).

**Table 2.** Comparison of six species of *Potamothrix* from Yunnan Lakes.

Species	*Potamothrix aductus* Cui & Wang, 2012	*Potamothrix parabedoti* sp. n.	*Potamothrix paramoldaviensis* sp. n.	*Potamothrix praeprostatus* sp. n.	*Potamothrix rhytipeniatus* Cui & Wang, 2012	*Potamothrix scleropenis* Cui & Wang, 2005
Hair chaetae	forward VII, plumose	forward III or V, plumose	absent	forward V, plumose	forward II, smooth	forward VI, plumose
Pectinate bifid chaetae	associated with hairs	associated with hairs	absent	associated with hairs	present	associated with hairs
Ventral chaetae	bifid	bifid	bifid	bifid, lower prong usually secondarily branched	bifid	bifid, lower prong usually secondarily branched
Spermathecal chaetae						
Penial chaetae	absent	absent	present	absent	absent	present
Length ration of vd/at	1:12-16	1:11-20	1:30-42	1:10-18	1:14-30	1:3
Prostate glands	present	absent	present	present	absent	absent
Male ducts	homogenous	homogenous	homogenous	homogenous	bipartite	homogenous
Penial sheath	absent	absent	absent	absent	absent	present
Habitats	Freshwater lake, 70-110 m depth, <15°C, fine clay	Freshwater lake, 70-120 m depth, <15 °C, fine clay.	Freshwater lake, 78 m depth, <16°C, fine clay	Freshwater lake, 113 m depth, < 14°C, fine clay	Freshwater lake, 5 m depth, 18°C, mud	Freshwater lake, 74 m depth, <15°C, fine clay

### Potamothrix parabedoti sp. n.

urn:lsid:zoobank.org:act:07854E46-F521-4B90-B580-D6862D494E1D

http://species-id.net/wiki/Potamothrix_parabedoti

#### Holotype.

IHB YAN 20021205c, mature specimen mounted in Canada balsam, and stained with borax carmine.

#### Type locality:

 IHB YAN20021205c, East of Lichang (24°32'04"N, 102°51'43"E) in Fuxian Lake, eastern Yunnan, China; depth 113 m, bottom temperature 13.5 °C, dissolved oxygen at bottom 5.2 mg/L, total nitrogen in water 0.164 mg/L, total phosphorus in water 0.037 mg/L, fine clay; Dec 11, 2002, coll. Y. Cui and X. Liu.

#### Paratypes:

 IHB YAN20021012b, East of Gushan Island (24°24'05"N, 102°52'45"E) in Fuxian Lake, eastern Yunnan, China; depth 76 m, bottom temperature 14.8 °C, dissolved oxygen at bottom 8.7 mg/L, total nitrogen in water 0.163 mg/L, total phosphorus in water 0.0203 mg/L, fine clay; Oct 8, 2002, coll. Y. Cui and X. Liu. IHB YAN20021009c, North of Dasazui (24°22'58"N, 102°49'49"E) in Fuxian lake, eastern Yunnan, China; depth 87 m, bottom temperature 14.7 °C, dissolved oxygen at bottom 8.7 mg/L, total nitrogen in water 0.165 mg/L, total phosphorus in water 0.022 mg/L, fine clay; Oct 8, 2002, coll. Y. Cui and X. Liu.

#### Etymology:

Named “*parabedoti*” for its resemblance with *Potamothrix bedoti* (Piguet, 1913) in terms of male genitalia.

#### Description:

 Two complete specimen 8.9–19.8 mm long (Holotype: 8.9 mm), with 36–131 segments (Holotype: 36), diameter at XI about 0.8 mm. Prostomium conical. Clitellum inconspicuous.

**Figure 3.**
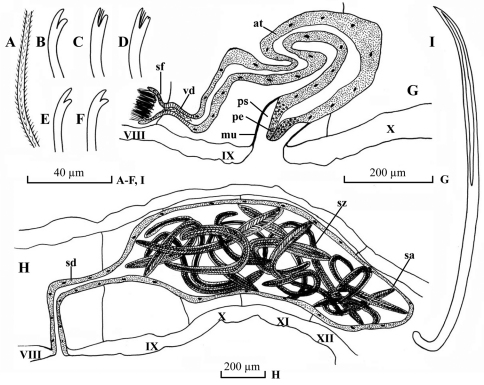
*Potamothrix paprbedoti* sp. n., **A** hair **B–D** distal end of dorsal bifids (VI, VII, XX, respectively**)E–F** distal end of ventral chaetae (II, XV, respectively) **G** lateral view of male duct in segments VIII–X **H** lateral view of spermatheca in segments VIII–XII **I** spermathecal chaeta. Scale bars: **A–F, I** 40 µm; **G–H2**00 µm.

Dorsal chaetae (Fig. 3B) of II (II–IV) bifid only, 7–8 per bundle, 100–145 µm long, 2.8–3.0 µm thick, upper tooth longer and thicker than lower. Dorsal bundle of III (V)-IX 4–8 hair chaetae and 5–8 bifid chaetae per bundle; plumose hair chaetae (Fig. 3A), 250–300 µm long, 2.8–3.2 µm thick basally; pectinate bifid chaetae (Fig. 3C–D), 140–150 µm long, 2.8–3.2 µm thick, with 1–3 intermediate teeth, upper tooth slightly longer and thinner than lower (usually bifurcated), or equally long. Dorsal bundles of posterior segments 2–4 hair chaetae and 3–4 bifid chaetae per bundle, shorter and thinner than those of anterior segments, hair chaetae 200–240 µm long, bifid chaetae 100–120 µm long, 2.6–2.8 µm thick. Ventral chaetae (Fig. 3E–F) bifid, 6–10 per bundle anteriorly, 100–150 µm long, 2.8–3.0 µm thick; 3–5 per bundle in postclitellar segments, 100–125 µm long, 2.4–2.6 µm thick, all with tooth similar to dorsal chaetae in II–IV. Spermathecal chaetae (Fig. 3I) one per bundle in middle to posterior of VIII or IX, entally embedded in glandular sacs, about 125–140 µm long, 4.0 µm thick, ental end strongly curved, with ectal part grooved. Penial chaetae absent. Male pores paired in line with ventral chaetae, middle to posterior of IX or X. Spermathecal pores paired in line with ventral chaetae, middle of X, immediately anterior to spermathecal chaetae.

Pharyngeal glands in II. Chloragogen cells from IV or V onwards. No coelomocytes. Male genitalia (Fig. 3G) paired. Vasa deferentia (Fig. 3G, vd) 45–70 µm long, 18–24 µm wide, entering atria apically. Atria (Fig. 3G, at) 880 µm long, 44–80 µm wide, tubular and rather homogenous throughout, with thin outer muscular layer and thick inner epithelium. Prostate gland absent. Soft part of penis (Fig. 3G, pe) cylindrical and tapering ectally, 80–100 µm long, basally 45–60 wide, ectally 25–36 wide, enclosed in penial sacs. Penial sac (Fig. 3G, ps) 80–130 µm long, 72–92 µm wide, with muscular layer 2–4 µm thick.

Spermathecae (Fig. 3H) in VIII–XIII or VIII, ducts (Fig. 3H, sd) 500–568 µm long, 74–95 µm wide, ampullae (Fig. 3H, sa) elongated, 470–1280 µm long, maximally 320–442 µm wide. Spermatozeugmata (Fig. 3H, sz) 10–25 in each ampulla, about 400–860 µm long.

#### Distribution and habitat:

Known only from its type locality, Yunnan Province, China; freshwater lake, 70–110 m depth, water temperature less than 15 °C, fine clay.

#### Remarks:

 According to short vasa deferentia, long tubular atria and lack of ejaculatory ducts, the new species fits more closely the definition of *Potamothrix* Vejdovský and Mrázek, 1902 than that of any other described tubificine genus (Brinkhurst and Jamieson 1971; Finogenova and Poddubnaja 1990).

The new speciesresembles *Potamothrix bedoti* (Piguet, 1913)in some aspects of reproductive organ (Vejdovský and Mrázek 1902), e.g. has a very short vasa deferentia, tubular atria without prostate gland, the length ration of vasa diferentia to atria, and the reproductive organs move to VIII–X. However, their differences are obvious. *Potamothrix parabedoti* sp. n., has homogenous atria with prostate glands, while *Potamothrix bedoti* has tripartite atria without prostate glands. In additional, the shapes of spermathecal chaetae are dissimilar in two of the species, the form is scalpel-like, and the ental part straight in *Potamothrix bedoti* (Timm 1970, 1999), but the ental end part is strongly curved in *Potamothrix parabedoti* sp. n (Fig. 3I).

The new species are distinguishable from other species from Yunnan Lakes in that of the position of their reproductive organs and the characteristic of some somatic chaetae. For instance, the reproductive organs are move to VIII–X in *Potamothrix parabedoti* sp. n. but that were in X–XIII in other species; the hair and pectinate bifids are absent in *Potamothrix rhytipeniatus* Cui & Wang, 2012 and *Potamothrix paramoldaviensis* sp. n., but present in other four species.

## Discussion

The principal distinguishable characteristics of the species of *Potamothrix* are given in Table 1 and Table 2. Nineteen previous species (Table 1) were divided into two groups, considered as subgenera, by lacking or possessing the prostate gland, respectively: *Potamothrix Potamothrix* Vejdovský & Mrázek, 1902 (type species: *Potamothrix moldaviensis* Vejdovský & Mrázek, 1902) and *Potamothrix Euilyodrilus* Brinkhurst, 1963 (type species: *Potamothrix hammoniensis* (Michaelsen, 1901) (Finogenova and Poddubnaja 1990)). Hence, six species of Yunnan lakes (Table 2), *Potamothrix scleropenis* Cui & Wang, 2005, *Potamothrix rhytipeniatus* Cui & Wang, 2012, and *Potamothrix parabedoti* sp. n., which lack prostate gland, belonged to the subgenus *Potamothrix*, and *Potamothrix aductus* Cui & Wang, 2012, *Potamothrix peniibristlatus* and *Potamothrix paramoldaviensis* sp. n., which possess prostate glands, belonged to the subgenus *Euilyodrilus*.

In the genus of *Potamothrix*, the histological structure of the epithelium of the atrium is taxonomically useful (Holmquist 1985; Finogenova and Poddubnaja 1990). According to histologically structure of the atrium, twenty previous species (Table 1) were divided into three types: (1) the ‘tripartite type’, which includes all the species with tripartite atrium, the short proximal part with densely granular inner epithelium layer, the long middle part with light granular inner epithelium, and the short distal part with homogenous inner layer (*Potamothrix alatus*, *Potamothrix bavaricus*, *Potamothrix bedoti*, *Potamothrix heuscheri*, *Potamothrix moldaviensis*, *Potamothrix thermalis*) (Brinkhurst and Jamieson 1971; Finogenova and Poddubnaja 1990; Milbrink and Timm 2001; Milbrink 1999; Timm 1970, 1999); (2) the ‘bipartite type’, comprising of the members with bipartite atrium, the short proximal part with densely granular inner epithelium layer and the long distal part with light granular inner epithelium (*Potamothrix caspicus*, *Potamothrix cekanovskajae*, *Potamothrix danubiali*, *Potamothrix hammoniensis*, *Potamothrix manus*, *Potamothrix marzeki*, *Potamothrix vejdovsky*) (Finogenova and Poddubnaja 1990; Hrabě 1981); and (3) the ‘homogenous type’, which consists of the taxa with homogenous atrium (*Potamothrix tudoranceai*, *Potamothrix postojnae*) (Šporka 1994; Brinkhurst and Wetzel 1984). Except the above mentioned 15 species, the histological of atrium of *Potamothrix prespaensis*, *Potamothrix isochaetus*, *Potamothrix orientalis*, and *Potamothrix ochridanus* was unmentioned in the original description (Brinkhurst and Jamieson 1971; Pop 1976), so that will need to be re-examined in the future. The species from Yunnan lakes excep *Potamothrix rhytipeniatus* are part of the ‘homogenous type’ (Table 2).

In addition, the presence of pectinate bifid chaetae accompanied with hair chaetae in the Yunnan lake species could be a special feature, but their position is variable. For instance, the hairs and pectinate bifids begin from segments V, VI, VII, III or V, respectively in *Potamothrix praeprostatus* sp. n., *Potamothrix scleropenis* Cui & Wang, 2005, *Potamothrix aductus* Cui & Wang, 2012,and *Potamothrix parabedoti* sp. n. The position of spermathecal pores of *Potamothrix* always lies in lateral line; however, in species from the Yunnan lakes, they were ventral instead of lateral.

As for habitat and distribution, the five species of *Potamothrix* from Fuxian Lake are well adapted to low dissolved oxygen concentrations, only found in the profundal region, to water depths lower than 70 m, water temperatures less than 16 °C, and they are found in sediments always clayey and sandy. Another species, *Potamothrix rhytipeniatus* Cui & Wang, 2012 was found in Xingyun Lake, in water depth of about 5 m, water temperature around 18 °C, and muddy sediments.

Lastly, according to some specific features, such as hair and pectinate bifid chaetae, spermathecal pore position, atrium histological structure, and their habitat, the species from Yunnan lakes maybe one new taxonomical group, the systematic placement of which needs further confirmation from more work.

### Key to the genus of Potamothrix Vejdovský and Mrázek, 1902

**Table:** 

1	Prostate glands present	2
–	Prostate glands absent	15
2	Hair chaetae present	3
–	Hair chaetae absent	11
3	With plumose hair chaetae	4
–	Without plumose hair chaetae	5
4	Prostate glands small, proximally attached to atria	*Potamothrix aductus* Cui & Wang, 2012
–	Prostate glands small, proximally attached to atria and far from vasa deferentia	*Potamothrix praeprostatus* sp. n.
5	Dorsal bifid chaetae pectinated	6
–	Dorsal chaetae bifid	9
6	Spermathecal chaetae present	7
–	Spermathecal chaetae absent	*Potamothrix postojnae* Karaman, 1974
7	Histological atria tripartite	8
–	Histological atria bipartite	*Potamothrix hammoniensis* (Michaelsen, 1901)
8	Length ratio of vasa deferentia to atria about 1:33–35	*Potamothrix alatus* Finogenova, 1972
–	Length ratio of vasa deferentia to atria about 1:20	*Potamothrix thermalis* (Pop, 1968)
9	Spermathecal chaetae present and modified	10
–	Spermathecal chaetae absent or 1–2 bifid chaetae	*Potamothrix ochridanus* (Hrabĕ, 1931)
10	Upper tooth of ventral chaetae just shorter or equal the lower	*Potamothrix vejdovsky* (Hrabĕ, 1941)
–	Upper tooth of ventral chaetae reduced	*Potamothrix svirenkoi* Lastočkin, 1937
11	Spermathecal chaetae present and modified	12
–	Spermathecal chaetae absent or 1–3 bifid chaetae	14
12	Penes present	13
–	Penes absent	*Potamothrix paramoldaviensis* sp. n.
13	Ventral chaetae 5–6 per bundle	*Potamothrix danubialis* (Hrabĕ, 1941)
–	Ventral chaetae 8–10 per bundle	*Potamothrix isochaetus* (Hrabĕ, 1931)
14	Upper tooth of bifid chaetae longer and thinner than the lower	*Potamothrix caspicus* (Lastočkin, 1937)
–	Upper tooth of bifid chaetae equal the lower	*Potamothrix marzeki* (Hrabĕ, 1941)
15	Hair chaetae present	16
–	Hair chaetae absent	23
16	With plumose hair chaetae	17
–	Without plumose hair chaetae	18
17	Male genitalia in X–XI, with penial sheath	*Potamothrix scleropenis* Cui & Wang, 2005
–	Male genitalia in VIII–IX, without penial sheath	*Potamothrix parabedoti* sp. n.
18	Dorsal bifid chaetae pectinated	20
–	Dorsal chaetae bifid	9
19	Upper tooth of ventral chaetae slightly longer and thinner than the lower	*Potamothrix orientalis* (Černosvitov, 1938)
–	Tooth of ventral chaetae equal in length	*Potamothrix prespaensis* (Hrabĕ, 1931)
20	Histological atria homogeneous	*Potamothrix tudoranceai* Šporka, 1994
–	Histological atria bipartite	*Potamothrix rhytipeniatus* Cui & Wang, 2012
–	Histological atria tripartite	21
21	Male genitalia in X–XI	22
–	Male genitalia in VIII–IX	*Potamothrix bedoti* (Piguet, 1931)
22	Length ratio of vasa deferentia to atria about 1:20	*Potamothrix heuscheri* (Bretscher, 1900)
–	Length ratio of vasa deferentia to atria about 1:8–9	*Potamothrix bavaricus* (Oschmann, 1913)
23	Spermathecal chaetae present and modified	24
–	Spermathecal chaetae absent or 4–5 bifid chaetae	*Potamothrix cekanovskajae* Finogenova, 1972
24	Histological atria bipartite	*Potamothrix manus* Finogenova, 1972
–	Histological atria tripartite	*Potamothrix moldaviensis* Vejdovský and Mrázek, 1902

## Supplementary Material

XML Treatment for Potamothrix

XML Treatment for Potamothrix praeprostatus

XML Treatment for Potamothrix paramoldaviensis

XML Treatment for Potamothrix parabedoti
